# Regional Consortia of Cancer Center Shared Resources: Models for Collaborative Infrastructure and the Emergence of a National Alliance

**DOI:** 10.7171/001c.168815

**Published:** 2026-09-09

**Authors:** Jay W. Fox, Gordon Roble

**Affiliations:** 1 Office of Research Core Administration University of Virginia Comprehensive Cancer Center; 2 Shared Resources Fred Hutchinson Cancer Center

**Keywords:** Shared Resources, Cancer Centers, Interinstitutional consortia, core facilities, NCI-designated Cancer Centers, resource sharing, collaborative infrastructure

## Abstract

Shared resource facilities are foundational to the scientific productivity of National Cancer Institute (NCI)-designated cancer centers, yet institutional silos frequently constrain member access to specialized instrumentation and expertise. Interinstitutional consortia of shared resources offer a pragmatic solution, enabling reciprocal service access at internal rates while preserving institutional autonomy. Here we describe two established regional consortia, the Mid-Atlantic Cancer Center Shared Resource Consortium (MACCSRC) and the Fred Hutchinson/University of Washington/Seattle Children’s NCI Comprehensive Cancer Center Consortium—examining their organizational frameworks, operational outcomes, and lessons learned. Finally, we introduce the recently organized National Alliance for Cancer Center Shared Resources and discuss how it may serve as an integrating infrastructure connecting regional networks on a national scale. Key considerations for consortium formation, including governance, intellectual property, pricing harmonization, and cost allocation, are addressed.

## Introduction

Shared resource facilities, also referred to as core facilities, are indispensable components of NCI-designated cancer centers. They provide member investigators with access to specialized instrumentation, technical expertise, and standardized workflows that would be prohibitively costly to replicate at the individual laboratory level. The operational and strategic management of these resources falls primarily to Associate Directors for Shared Resource Management (AD-SRMs), whose portfolios encompass service delivery, equipment stewardship, cost recovery, and long-range planning.

In professional forums, including the Association of Biomedical Resource Facilities (ABRF) annual meeting and regional chapter gatherings, AD-SRMs consistently meet and discuss a recurring set of challenges: gaps in local service coverage, instrument downtime, uneven utilization, and the high capital cost of emerging technologies. These conversations have catalyzed informal peer networks and, in some cases, formalized interinstitutional agreements designed to extend service access beyond individual institutional boundaries.

In fluctuating times of scientific funding and shifting priorities, shared resources are often early sentinels of real change based on investigator demand and usage and are also often the early developers of practices to best manage changing priorities to continue supporting investigators.^1,2^ Consortia working together, sharing best practices, and supporting each other’s investigators as necessary further strengthen the ability to weather changes in funding and policies.

The present commentary describes the organizational history, governance structure, and operational experience of two distinct consortium models: the Mid-Atlantic Cancer Center Shared Resource Consortium (MACCSRC), which is one of the earliest formal regional consortia of NCI-designated cancer centers; and the long-standing Fred Hutchinson/University of Washington/Seattle Children’s NCI Comprehensive Cancer Center Consortium, which has more recently undertaken systematic harmonization of shared resource access and pricing. Together, these examples illustrate how interinstitutional collaboration can take different forms depending on geographic, institutional, and historical context. We also relate these efforts in the context of the newly established National Alliance for Cancer Center Shared Resources.

## The Mid-Atlantic Cancer Center Shared Resource Consortium (MACCSRC)

### Origins and Rationale

The MACCSRC emerged organically from collegial interactions among AD-SRMs of four NCI-designated cancer centers in the Mid-Atlantic region: the University of Virginia (UVA) Comprehensive Cancer Center, the University of Maryland Marlene and Stewart Greenebaum Comprehensive Cancer Center, Georgetown University Lombardi Comprehensive Cancer Center, and the Johns Hopkins University Sidney Kimmel Comprehensive Cancer Center. These individuals crossed paths repeatedly while serving on external advisory committees and at professional meetings, and their discussions consistently centered on common operational challenges.

The founding proposition for a consortium was straightforward. Each member cancer center would make its full portfolio of shared resource services available to cancer center members at all other member institutions, offering the services at the same internal rate applicable to home institution members, without the addition of facilities and administrative costs. The anticipated benefits were threefold. First, the arrangement would expand access to specialized instrumentation and services not locally available at each institution. Second, investigators would benefit from member-equivalent pricing, which is typically more favorable than rates available from nonmember or commercial providers. Third, and critically, the involvement of the host institution’s AD-SRM as a point of contact would provide accountability and ensure timely, high-quality sample processing — an assurance difficult to obtain through arms-length service agreements.

On this basis, the founding AD-SRMs, Dr. Jay Fox (UVACCC), Dr. Nick Ambulos (University of Maryland Greenebaum), Dr. Stephen Beyers (Georgetown Lombardi), and Mr. Jeff Smith, MBA (Johns Hopkins Sidney Kimmel), proposed the formal establishment of the MACCSRC, with the group to be governed by a memorandum of understanding (MOU) among member institutions.

### Governance, MOU Negotiation, and Institutional Considerations

While the concept garnered broad enthusiasm among cancer center directors and scientific staff, formalizing the arrangement through an institutional MOU proved to be more complex. Concerns raised by institutional representatives centered on three primary issues: (1) intellectual property (IP) ownership and the potential for proprietary data to be exposed during cross institutional sample processing; (2) proportional cost allocation and the equitable distribution of financial obligations and benefits across member institutions of varying size; and (3) perceived risks to institutional identity and operational autonomy.

Resolution of these concerns required sustained dialogue among AD-SRMs, cancer center directors, and senior institutional leadership. These conversations were essential for clarifying both the scope and the boundaries of the consortium, specifically, that the arrangement was designed to supplement rather than supplant local services and that no institutional identity would be subsumed by membership. Negotiations spanned approximately 12 months before all parties reached an agreement, and the MOU was executed in 2016.

This experience highlights a generalizable principle: the social and administrative infrastructure required for consortium formation is often more time-intensive than the logistical infrastructure, and early engagement of institutional stakeholders, including research offices, legal counsel, and finance teams, is essential to a timely and durable agreement.

### Operational Outcomes Over a Decade of Collaboration

Since its inception, the MACCSRC has tracked interinstitutional commerce among member shared resources. The patterns of utilization have confirmed the founding hypotheses while also providing important nuance. Notably, no member institution has elected to discontinue a local service in favor of outsourcing it to a consortium partner. This finding validates the original premise that the consortium supplements rather than displaces local capacity.

Observed drivers of cross institutional utilization fall into four primary categories: (1) temporary or sustained lack of local capacity, including instrument downtime; (2) absence of a specific service or technology at the home institution; (3) availability of a complementary instrument or methodology at a partner institution; and (4) prospective evaluation of demand before local service launch. This last use case merits particular attention: Accessing a partner’s service over time allows an AD-SRM to assess whether sufficient local investigator demand exists to justify capital investment, operational costs, and staffing requirements associated with establishing a new core service.

Beyond direct service sharing, the consortium has demonstrated value in National Institute of Health (NIH) Shared Instrumentation Grant (S10) applications. When a member institution submits an S10 application, partner AD-SRMs have provided letters of support documenting cross institutional need for the requested instrument, which strengthens applications by demonstrating broader access and utilization across a research community that extends beyond a single campus.

### Membership Growth and Network Sustainability

Since its founding, the MACCSRC has expanded to include two additional NCI-designated cancer centers: the Hollings Cancer Center at the Medical University of South Carolina, which joined in 2018 and the Massey Comprehensive Cancer Center at Virginia Commonwealth University, which joined in 2026. The geographic footprint of the consortium now spans the Mid-Atlantic and Southeastern United States, reflecting the scalability of the MOU-based governance model.

All current member institutions report satisfaction with the consortium and affirm their ongoing commitment to its mission. This sustained engagement over nearly a decade is itself a meaningful indicator of the consortium’s operational and strategic value, particularly given the pressures on shared resource budgets and the high rate of leadership turnover characteristic of academic medical environments.

## The Fred Hutchinson/University of Washington/Seattle Children’s NCI Comprehensive Cancer Center Consortium

### Background and Consortium Structure

The Fred Hutchinson Cancer Center/University of Washington (UW)/Seattle Children’s Research Institute (SCRI) consortium was designated as an NCI Comprehensive Cancer Center Consortium in 1973, making it one of the longest-standing multi-institutional cancer center partnerships in the United States. Despite the durability and scientific strength of this collaboration, most shared resources have historically been centralized at Fred Hutchinson, creating practical inequities in access that persisted even across geographic distances of less than five miles. Over the past three years, focused institutional efforts have addressed these challenges through two principal strategies: pricing harmonization and the deliberate expansion of shared resource capabilities across all partner sites.

From an administrative standpoint, consortium members at UW and SCRI access Fred Hutchinson shared resources through standardized MOUs established between institutional pairs (i.e., Fred Hutch–UW and Fred Hutch–SCRI). These agreements define consistent terms and conditions, including requirements for active purchase orders tied to shared resource work orders. At Fred Hutchinson, these processes are centrally managed by the Shared Resources Administration team, with iLab Solutions serving as the primary platform for usage tracking, billing, and reporting.

### Pricing Harmonization and Its Impact on Utilization

A key recent initiative has been the implementation of a unified pricing structure that provides consortium members, egardless of institutional affiliation, the same rates as internal Fred Hutchinson investigators. This approach ensures that Cancer Center Support Grant (CCSG) funding benefits all consortium members equitably and expands access to specialized equipment not available at every partner site.

Critically, this pricing model has not resulted in revenue loss. On the contrary, harmonized pricing has increased utilization across nearly all 12 Fred Hutchinson shared resources. While the absolute number of new consortium users remains modest, existing consortium users have substantially increased their utilization, resulting in a disproportionate increase in overall revenue (Figure 1). This pattern suggests that pricing barriers, rather than lack of scientific need, were the primary constraint on cross institutional utilization before harmonization.

**Figure 1.**
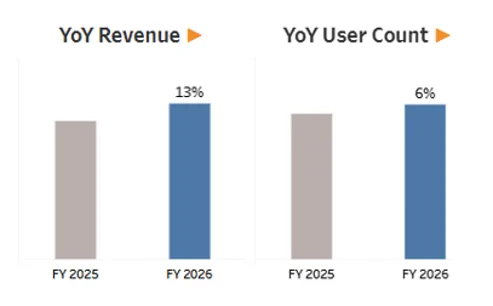
Year-over-year growth in revenue and user counts across Fred Hutchinson Shared Resources**. This growth is attributed to consortium utilization for the same 10-month period in the fiscal years 2025–2026.

### Complementary Instrumentation and Coordinated Service Delivery

Strong communication and coordination within shared resource leadership have created meaningful operational synergies, enabling more strategic and complementary capital investments in instrumentation. A paradigmatic example is the coordinated acquisition of next-generation sequencing platforms. Fred Hutchinson installed an Illumina NovaSeq X Plus in its genomics shared resource (2024), while SC acquired a complementary long-read sequencing platform, the PacBio Revio in the same year. Although each instrument is operated independently by its host institution, sample preparation can occur at either site, with coordinated pipelines for sample transport and data delivery. Users experience a seamless service regardless of where analysis is performed. This model has increased utilization of both platforms while avoiding redundant capital investments in the six- to seven-figure range.

Similar strategies have been applied to high-cost, historically underutilized technologies, including light-sheet microscopy and advanced imaging systems. Each institution facilitates cross institutional access by granting nonemployee affiliate status to qualified users from partner organizations. Following institution- and resource-specific training, affiliated users access equipment using the same reservation systems and receive the same scheduling prioritization as internal investigators (Figure 2).

**Figure 2.**
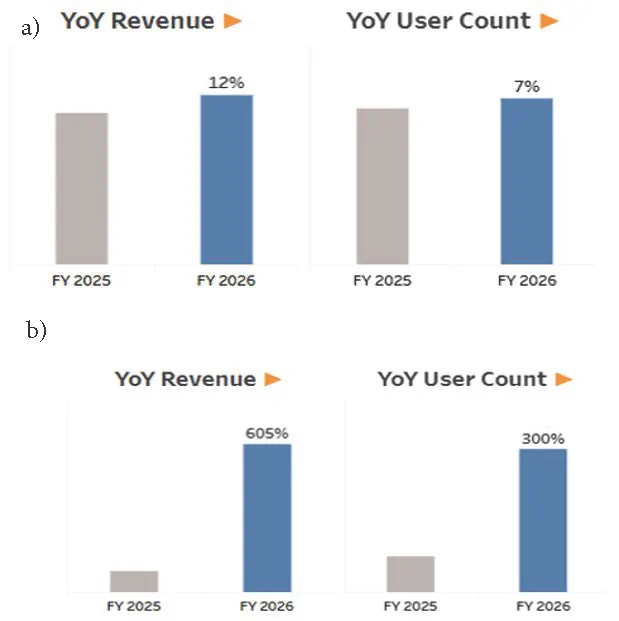
Year-over-year growth in revenue and user counts for (a) the Fred Hutchinson Genomics and Bioinformatics Shared Resource and (b) Cellular Imaging Shared Resource.** This growth is attributed to consortium utilization for the same 10-month period in each of the fiscal years 2025–2026.

### Regulatory Infrastructure and Preclinical Research Collaboration

The collaborative framework extends to the regulatory infrastructure supporting preclinical animal research. UW houses the consortium’s only PET/CT and precision radiation therapy systems. At the same time, Fred Hutchinson maintains a cryogen-free small animal MRI with both 7T and 3T capabilities, a platform valued for its translational alignment with clinical human imaging systems. Through interinstitutional Institutional Animal Care and Use Committee (IACUC) agreements and shared umbrella protocols, investigators can conduct studies across sites with minimal administrative burden. Animal transport services, provided through each institution’s comparative medicine program, ensure full compliance with applicable ethical and regulatory standards.

### Collaborative S10 Grant Strategy

The consortium’s collaborative model has also strengthened NIH Shared Instrumentation Grant (S10) applications. Proposals are led by the best positioned institution based on demonstrated scientific need and technical expertise, with consortium partners providing coordinated support through letters of collaboration, identification of major and minor users, and contributions to scientific justification sections. Consortium investigators also participate in instrument advisory committees, which broaden the governance and accountability structures associated with awarded instruments. Collectively, this collaborative approach has contributed to five successful S10 awards over the past three years.

## Comparative Analysis: Two Models of Interinstitutional Collaboration

Although both the MACCSRC and the Fred Hutch/UW/SC consortium represent successful models of interinstitutional shared resource collaboration, they differ meaningfully in their origins, governance structures, and operational emphases. These differences reflect the distinct institutional and geographic contexts from which each emerged.

The MACCSRC was constituted de novo as a voluntary network among previously independent cancer centers, requiring the establishment of new MOU frameworks and considerable negotiation to align institutional interests. Its primary mechanism is reciprocal service access at internal rates, and its value is most evident in scenarios of local capacity gaps, instrument downtime, and exploratory demand assessment. The consortium spans multiple states and operates without centralized administrative infrastructure, relying instead on collegial relationships among AD-SRMs.

The Fred Hutch/UW/SCRI consortium, by contrast, operates within a pre-existing NCI-designated consortium structure that has evolved over five decades. Its recent advances, including pricing harmonization, coordinated instrumentation acquisition, and cross institutional affiliate access, represent a maturation of an already formalized partnership. The geographic proximity of the three institutions, combined with centralized administrative support at Fred Hutchinson, enables a higher degree of operational integration, including coordinated sample transport, shared regulatory protocols, and joint S10 strategies.

Together, these models illustrate that effective shared resource collaboration does not require a single organizational template. The key enabling factors, trust among leadership, clearly defined governance, internal pricing access for partners, complementary (rather than redundant) investments, and coordinated grant strategy, are transferable across diverse institutional contexts.

## The National Alliance for Cancer Center Shared Resources: Toward a Federated Infrastructure

The success of the MACCSRC, the Fred Hutch/UW/SC consortium, and other analogous regional networks has informed the establishment of the National Alliance for Cancer Center Shared Resources (NACCSR). The National Alliance is conceived as an integrating infrastructure that can connect regional networks, facilitate the exchange of best practices, and collectively address systemic challenges facing cancer center shared resources on a national scale.

The National Alliance is not designed to supersede regional networks, but rather it serves as a coordinating body that amplifies their collective impact. Potential functions include: harmonizing MOU templates and governance frameworks to facilitate the formation of new regional consortia; creating a national registry of available shared resource services and instrumentation to support cross regional referrals; advocating for policies that recognize and incentivize interinstitutional resource sharing at the federal level; and providing a professional home for AD-SRMs engaged in collaborative resource management.

## Conclusions and Key Considerations for Consortium Formation

The experience of both the MACCSRC and the Fred Hutch/UW/SC consortium demonstrates that formal interinstitutional collaboration in shared resource management is both feasible and durable across a range of organizational contexts. The following considerations are offered to institutions contemplating the formation or expansion of similar networks:

- **Engage institutional stakeholders early.** Legal, finance, and research administration offices must be involved from the outset to identify and resolve concerns related to IP, cost allocation, and liability before momentum is lost.
- **Define scope explicitly.** Governing agreements should clearly articulate that the consortium supplements and does not replace local service capacity, and that institutional autonomy over local operations is preserved.
- **Harmonize pricing proactively.** Extending internal rates to consortium partners removes a major barrier to utilization and, as the Fred Hutch/UW/SC experience demonstrates, typically increases rather than reduces overall revenue.
- **Invest in complementary rather than redundant instrumentation.** Coordinated capital planning across consortium members maximizes collective coverage while avoiding duplicative expenditures.
- **Designate accountable points of contact.** The assignment of AD-SRMs as institutional liaisons ensures service quality and turnaround standards are maintained across institutional boundaries.
- **Leverage consortium membership in grant applications.** S10 applications benefit from documented cross institutional need, and consortium partners can provide substantive letters of support and user data.
- **Track utilization systematically.** Longitudinal data on interinstitutional commerce support ongoing evaluation of consortium value and provide evidence for grant applications and institutional reports.

As the landscape of cancer research becomes increasingly technology-intensive and capital-constrained, interinstitutional resource sharing represents a strategically rational response to the limitations of siloed institutional models. The models described here whether constituted as voluntary regional consortia or as formalized NCI-designated partnerships demonstrate that collaborative investment in shared infrastructure enhances scientific capacity, equity of access, and long-term sustainability. Regional consortia, such as the MACCSRC and the Fred Hutch/UW/SC consortium, supported by a nascent national infrastructure through the National Alliance for Cancer Center Shared Resources, offer proven paths toward more equitable and efficient access to the shared resources that underpin cancer center science.
